# The direction and rate of spread of chronic wasting disease

**DOI:** 10.3389/fvets.2026.1827624

**Published:** 2026-07-08

**Authors:** Arun Suresh, Zoe Shanley, Shane Hesting, Levi Jaster, Akila Raghavan, Ram K. Raghavan

**Affiliations:** 1 Department of Mathematics, College of Arts and Sciences, University of Missouri, Columbia, MO, United States; 2 Kansas Department of Wildlife and Parks, Emporia, KS, United States; 3 Department of Pathobiology and Integrative Biomedical Sciences, College of Veterinary Medicine, University of Missouri, Columbia, MO, United States; 4 Department of Public Health, College of Health Sciences, University of Missouri, Columbia, MO, United States; 5 MU Institute for Data Science and Informatics, University of Missouri, Columbia, MO, United States

**Keywords:** chronic wasting disease, direction of spread, Kansas, rate of spread, mathematical modeling

## Abstract

**Introduction:**

Chronic wasting disease (CWD) is a fatal transmissible prion disease of cervids that continues to expand across North America. Although the spatiotemporal distribution of CWD in the central United States has been extensively documented, the direction and rate of disease spread remain poorly understood, limiting the implementation of proactive surveillance and mitigation strategies.

**Methods:**

We developed a data-driven spatiotemporal modeling framework to quantify the direction and rate of CWD spread across Kansas using surveillance data collected between 2005 and 2023. Kansas was partitioned into 20 km^2^ spatial grid cells, and disease dynamics within each cell were modeled using a system of differential equations representing susceptible, infected, and environmental compartments. Spatial migration processes were incorporated through a stochastic mixing matrix informed by empirically observed patterns of first infection among neighboring cells. Model parameters were optimized through a grid search of plausible values derived from published literature, and predictive performance was evaluated using receiver operating characteristic (ROC) analysis. To characterize spread dynamics, we applied a weighted centroid approach at the zonal scale and a migration-based vector flow analysis at the local scale.

**Results:**

The model successfully reproduced the observed spatiotemporal progression of CWD across Kansas with high predictive accuracy (mean AUC = 0.88). Zonal analyses revealed a predominant northwest-to-southeast progression of infection, with substantial regional heterogeneity in both direction and velocity of spread. Local vector-flow analyses identified multiple transmission corridors and persistent hotspots that may function as regional sources of infection dissemination. Rates of spread quantified at both zonal and local scales corroborated the directional trends observed in the flow analyses and highlighted areas characterized by elevated transmission intensity and migratory spread. Importantly, the model demonstrated strong forecasting capability by anticipating emerging areas of infection prior to their confirmation through surveillance.

**Discussion:**

By integrating mechanistic disease dynamics with empirically informed migration processes, our framework provides a quantitative characterization of both the direction and rate of CWD spread at two spatial scales. These findings offer actionable insights for wildlife disease management by supporting targeted surveillance, boundary monitoring, and region-specific intervention strategies.

## 1 Introduction

Chronic wasting disease (CWD) is a progressive, fatal neurodegenerative disease affecting cervids in North America, Scandinavia, and South Korea, and since its first identification in 1967 at a research facility in Colorado, CWD has expanded steadily across large portions of the United States and Canada (1, 2). The disease is caused by misfolded prion proteins that accumulate in neural and lymphoid tissues and persist in the environment, facilitating indirect transmission long after infected animals have died. At present, no vaccine or curative treatment exists for CWD or other transmissible spongiform encephalopathies, and disease management relies primarily on surveillance, movement restrictions, and localized interventions such as targeted culling, approaches that vary widely among jurisdictions and are not universally permitted. Kansas, the geographic focus of this study, exemplifies these challenges (3, 4). Targeted culling is not authorized in the state, and effective large-scale management options to prevent further spread are limited.

CWD primarily affects white-tailed deer (*Odocoileus virginianus*) and mule deer (*O. hemionus*), two species of substantial ecological and economic importance. Recreational hunting of these species contributes significant revenue to rural economies throughout the Midwest (4, 5). Beyond economic impacts, sustained transmission of CWD poses a risk of long-term population declines, as documented in regions such as Wyoming (6, 7). Consequently, there is strong interest among wildlife managers and policymakers in understanding not only where CWD is present, but also how it moves across landscapes and how rapidly new areas become affected.

Our previous work have characterized the spatiotemporal distribution of CWD in Kansas and identified environmental and demographic factors associated with disease occurrence (3, 4). These studies suggested a general pattern of expansion from the northwest toward the eastern and southeastern regions of the state. However, the directionality and rate of this expansion have not been formally quantified for this region or, to our knowledge, for other endemic areas. Such metrics are essential for anticipating future disease fronts, optimizing surveillance design, and implementing regionally targeted management strategies.

Estimating the movement of wildlife diseases across space has been addressed in other systems using approaches such as dynamic occupancy models [e.g., (8, 9)] and related spatial diffusion frameworks [e.g., Wang et al. (10)]. While these methods have provided valuable insights, they often rely on annual snapshots of disease presence and may be limited in their ability to capture continuous spatial dynamics or account for sparse detection data, an important consideration for CWD, where early detection is difficult because of long incubation periods and subclinical infections. Additionally, wildlife surveillance datasets are typically highly outcome-imbalanced, with relatively few positive detections compared with samples that test negative for the presence of a disease condition.

To address these challenges, we developed a spatiotemporal modeling framework that integrates mechanistic disease dynamics with empirically informed spatial migration. Our approach coupled a compartmental model of CWD transmission within spatial units with a migration matrix derived from observed patterns of first detection across neighboring areas. This structure allowed us to simulate disease progression continuously in time while explicitly accounting for cervid movement between adjacent regions. By calibrating the model to long-term surveillance data from Kansas, high-resolution estimates of CWD prevalence were generated in both space and time, including in areas where direct observations were limited. This framework enabled two complementary analyses of disease spread. At a regional scale, we tracked weighted centroids of simulated infection-presence to quantify the dominant direction and average velocity of CWD movement across predefined zones. At a local scale, we employed the migration structure of the model to estimate instantaneous flow vectors between neighboring spatial grid cells, revealing fine-scale transmission pathways and potential corridors of spread. Together, these analyses provided a quantitative assessment of the rate and direction in which CWD is moving across Kansas.

The objective of this study was therefore to characterize the rate and direction of CWD spread across Kansas using a unified spatiotemporal modeling approach. By producing both global and local measures of disease movement, actionable insights can be derived to inform surveillance design, identify emerging hotspots, and support evidence-based wildlife disease management in systems where conventional interventions remain limited.

## 2 Materials and methods

### 2.1 Surveillance data

**Figure 1:**
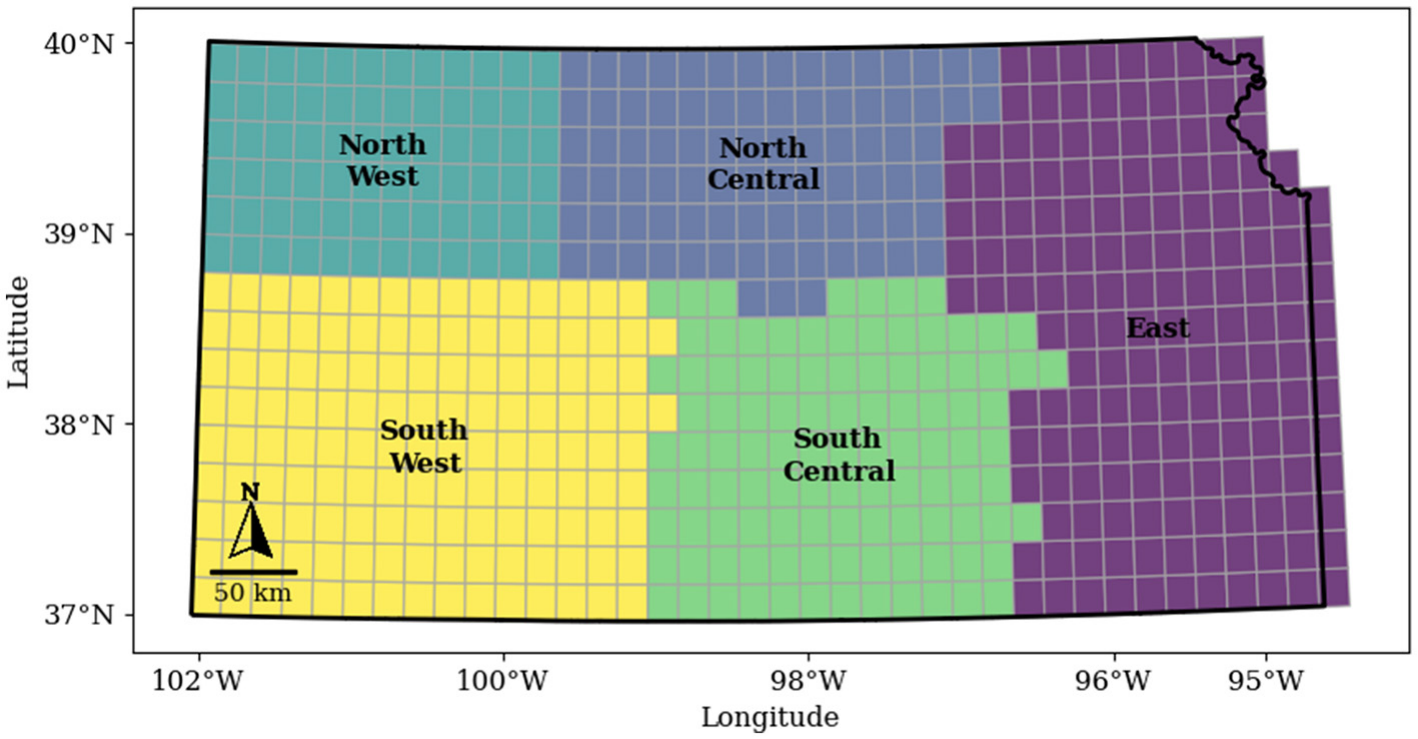
The five chronic wasting disease management zones in Kansas, and the 20 km^2^ grid cells used as spatial units of analysis in the study.

We analyzed statewide chronic wasting disease (CWD) surveillance data collected in Kansas from 2005 to 2023. Each record corresponded to an individual cervid sampled through hunter harvest, targeted surveillance, or other wildlife monitoring programs and included test results for CWD infection (4). To support spatial modeling, Kansas was partitioned into a uniform grid comprising a total of 646 spatial units (henceforth cells), each of dimension 20 × 20 km that tile the state (Figure 1). Each sample was assigned to its corresponding cell based on geographic location. For each cell, we aggregated annual counts of CWD-positive detections and determined the first year in which infection was recorded. We adopted a “once-positive-always-positive” (OPAP) assumption based on the current knowledge of CWD persistence among cervid populations and in the environment, combined with limited management options, i.e., once a positive CWD detection was recorded from a sample originating from cervids in a cell, it was treated as persisting thereafter.

### 2.2 Mathematical modeling

#### 2.2.1 Spatial grid

We constructed a 17 × 38 rectangular grid of cells with dimensions 20 × 20 km each, tiling the entire state of Kansas (Figure 1). For modeling purposes, the cells were indexed 1, …, 646, thereby allowing cell-level quantities to be represented as vectors in ℝ^646^. Furthermore, we assigned each cell a label representing its associated management zone, established by the Kansas Department of Wildlife and Parks (KDWP) (11). A cell intersecting a zonal boundary was assigned to the zone that covered the greatest proportion of its area.

#### 2.2.2 Temporal component

To model the temporal dynamics of disease evolution within cell boundaries, we adapted the system of differential (Equations 1) introduced by Sharp and Pastor (12), which captures the temporal evolution of susceptible and infected populations alongside the growth and decay of prion mass in the environment:


$$\begin{array}{l} \frac{dS}{dt}=S\left[r\left(1-\frac{S+I}{k}\right)-\beta E\right]\\ \;\frac{dI}{dt}=\beta SE-\mu I\\ \frac{dE}{dt}=\epsilon I-\tau E \end{array}$$ (1)

where *S*(*t*) and *I*(*t*) denote the respective sizes of the susceptible and infected cervid population within a cell at time *t*, and *E*(*t*) denotes the mass of infectious material present within the cell at time *t*. The first equation follows a logistic growth trend of the susceptible population with per-capita birth rate *r*, assuming the carrying capacity of the cell is *k*. This growth is offset by susceptible cervids encountering other infected cervids in the area, with β being the indirect transmission coefficient. The second equation describes the accumulation of infected cervids through exposure and their removal through infection-induced mortality, with mortality rate μ. The third equation describes deposition of infectious material at rate ϵ and its loss/decay at rate τ. The parameter definitions and their corresponding units are provided in Table 1. By adapting this model to our problem, we assumed that transmission primarily occurs through direct deer-to-deer contact and/or environmental exposure, an assumption that is consistent with prior work identifying these pathways as the dominant mechanisms of CWD persistence and spread (13–15).

**Table 1:** Parameters of model (1) and their units of measurement.

Parameter	Definition	Units
*r*	Per-capita birth rate of deer	time^−1^
*k*	Environmental carrying capacity (per cell)	number
β	Indirect transmission coefficient	mass^−1^ time^−1^
μ	Death rate of infected animals	time^−1^
ϵ	Per-capita rate of excretion of infectious material	time^−1^
τ	Mass-specific loss of infectious material	time^−1^

#### 2.2.3 Spatial component (migration)

We incorporated a spatial component into our temporal model, representing a constant rate of migration between cells, using a spatial mixing matrix *M* ∈ ℝ^646 × 646^, following methods introduced by Johns and Mehl (16). Migration was assumed to occur only between neighboring cells, an assumption that is consistent with observed deer movement behavior (17) and commonly adopted in dynamic occupancy and spatial epidemiological models (3, 18, 19).

For each pair of distinct cells *i* and *j*, the (*i, j*)−th entry of *M*, denoted by *m*_*i* ← *j*_, represents the proportion of infected cervids migrating from cell *j* to cell *i*. If cells *i* and *j* are not neighbors, we set *m*_*i* ← *j*_ = 0. For neighboring cells *i* and *j* (henceforth written *i* ~ *j*), the migration proportions *m*_*i* ← *j*_ were defined as a combination of two components: a data-informed migration component *p*_*i* ← *j*_ to explain the observed migration patterns from the dataset and a stochastic component η_*i* ← *j*_ to account for unobserved variability in migration.

We determined the data-informed component *p*_*i* ← *j*_ between neighboring cells as follows: for each *i* = 1, …, 646, we determined from data, the year *y*_*i*_ in which infection was first detected in cell *i*. If such a year does not exist for a given cell, we set *p*_*i* ← *j*_ = 0. Let *d*_*i*_ denote the number of infectious samples detected in cell *i* in year *y*_*i*_. For each neighbor cell *j* of *i*, we defined *d*_*j*_ to be the total number of infected samples observed in cell *j* between the years 2005 and *y*_*i*_ − 1. Under the OPAP assumption, the values *d*_*j*_ provided an estimate on the number of infectious cervids known to be present in cell *j* during year *y*_*i*_. With these definitions in place, we defined *p*_*i* ← *j*_ to be:


$$\begin{array}{l} p_{i\leftarrow j}=\frac{d_{j}}{d_{i}+\sum_{k\sim i}d_{k}} \end{array}$$ (2)

Viewed differently, for each *i*, the collection {_*p*_*i*←*k*_}*k*~*i*_ as defined in Equation 2 above, gives a probability distribution. More precisely, given that infection is detected in cell *i* in year *y*_*i*_, the value of *p*_*i* ← *j*_ determines the probability (indicated by the data) that infection was seeded into cell *i* through cell *j*. Since the data-informed proportions *p*_*i* ← *j*_ can only be as good a measure of migration as the underlying data, we incorporated a stochastic component to our migration proportions by augmenting *p*_*i* ← *j*_ with a noise term η_*i* ← *j*_ ~ *Unif* (0, 1). Altogether, Equation 3 gives the migration proportions *m*_*i* < −*j*_ as:


$$\begin{array}{l} m_{i\leftarrow j}=\frac{p_{i\leftarrow j}+\eta_{i\leftarrow j}}{2n_{i}} \end{array}$$ (3)

where *n*_*i*_ represents the number of neighbors of cell *i*. With all these notations in place, we defined the migration matrix *M* as:


$$\begin{array}{l} M_{ij}=\{\begin{array}{l} m_{i\leftarrow j}\;i\neq j\\ 1-\underset{k\sim i}{\sum }m_{k\leftarrow i}\;i=j\; \end{array} \end{array}$$ (4)

and verified that the diagonal elements of *M* are always non-negative, thereby confirming that *M* is indeed a left-stochastic matrix by design. This ensured that the total population mass is conserved during migration. The following example illustrates how *M* encodes the migratory patterns of cervids across cells.

**Example:** in this example, let us consider a simpler setup consisting of three cells labeled 1, 2 and 3, arranged in the following configuration:

**Figure:**
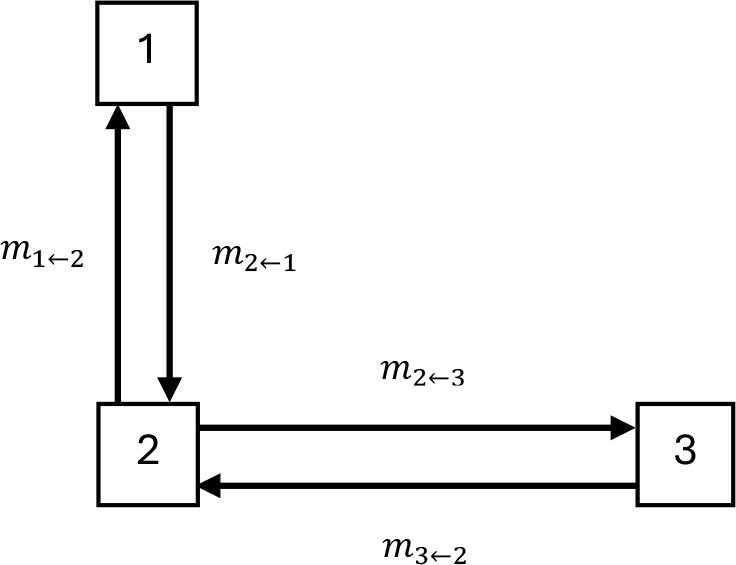


Let us also assume that these migration proportions *m*_*i* ← *j*_ have been determined. The migration matrix defined in Equation 4 takes the following form when adapted to this case


$$\begin{array}{l} M=\left[\begin{array}{ccc} 1-m_{2\leftarrow 1} & m_{1\leftarrow 2} & 0\\ m_{2\leftarrow 1} & 1-\left(m_{1\leftarrow 2}+m_{3\leftarrow 2}\right) & m_{2\leftarrow 3}\\ 0 & m_{3\leftarrow 2} & 1-m_{2\leftarrow 3} \end{array}\right] \end{array}$$

To see how *M* encodes the dynamics of migration, let us suppose that at time *t* = 0, the infection numbers across the three cells were determined to be $I\left(0\right)={\left[I_{1},I_{2},I_{3}\right]}^{T}\in {\mathbb{R}}^{3}$. Ignoring any temporal effects, we see that after a discrete (Euler) time-step *t*: 0 → 1, the new infection numbers post-migration can be obtained from *I*(0) as follows:


$$\begin{array}{l} I\left(1\right)=M\;I\left(0\right)\\ =\;\left[\begin{array}{ccc} 1-m_{2\leftarrow 1} & m_{1\leftarrow 2} & 0\\ m_{2\leftarrow 1} & 1-\left(m_{1\leftarrow 2}+m_{3\leftarrow 2}\right) & m_{2\leftarrow 3}\\ 0 & m_{3\leftarrow 2} & 1-m_{2\leftarrow 3} \end{array}\right]\;\left[\begin{array}{c} I_{1}\\ I_{2}\\ I_{3} \end{array}\right]\\ =\;\left[\begin{array}{c} \left(1-m_{2\leftarrow 1}\right)I_{1}+m_{1\leftarrow 2}I_{2}\\ m_{2\leftarrow 1}I_{1}+\left(1-m_{1\leftarrow 2}-m_{3\leftarrow 2}\right)I_{2}+m_{2\leftarrow 3}I_{3}\\ m_{3\leftarrow 2}I_{2}+\left(1-m_{2\leftarrow 3}\right)I_{3} \end{array}\right] \end{array}$$

where, for instance, the first component of *I*(1) captures two different ways an infection persists in cell 1, namely via the infectious animals (1 − *m*_2←1_)*I*_1_ that did not migrate during this time-step, and additionally via an influx *m*_1←2_*I*_2_ of new infectious animals migrating into cell 1 from cell 2. The other two components can be interpreted similarly.

#### 2.2.4 Spatiotemporal model

To define our coupled spatiotemporal model, we began by letting *S*(*t*), *I*(*t*) ∈ ℝ^646^ denote the number of susceptible and infected cervids in each cell at time *t*, and *E*(*t*) ∈ ℝ^646^denote the mass of infectious material present in each cell at time *t*. With these in place, the resulting spatiotemporal model is given by:


$$\begin{array}{l} \frac{dS}{dt}=S⊙\left[r\left(1-\frac{S+I}{k}\right)-\beta E\right]+MS\\ \frac{dI}{dt}=\beta S⊙E-\mu I+MI\\ \frac{dE}{dt}=\epsilon I-\tau E \end{array}$$ (5)

where ⊙ denotes the Hadamard (elementwise) product of vectors. The model defined in Equation 5 augments the localized temporal evolution of the infection with a dynamic spatial component that simulates migratory behavior.

#### 2.2.5 Model initialization and parameter estimation

To numerically integrate (5), we initialized *S*(0), *I*(0) and *E*(0). Environmental contamination *E*(0) was initialized as a random vector with entries drawn from a standard normal distribution. The initializations *S*(0) and *I*(0) were obtained by aggregating susceptible and infected counts within each cell for a suitable simulation start year *t*_0_.

To test model sensitivity to initial conditions, three separate starting years *t*_0_ were considered from a period preceding the longest contiguous sequence of years exhibiting substantial spatial spread in the number of infected cells. Letting *t*_1_ denote the last year of this longest contiguous sequence, we defined the interval [*t*_0_, *t*_1_] to be the primary validation window. In addition, to reduce run-to-run variability introduced by the stochastic noise component, we conducted 50 independent trials for each of the three possible starting years. For each of these trials, the optimal values of the parameters in (5) were determined through a grid-search guided by previously published estimates. The per-capita birth rate was chosen from r ε {1.5, 1.75, 2.0, 2.25, 2.5} based on demographic estimates in Green et al. (20) for Midwestern white tailed deer, the dominant species in our dataset. We determined the environmental carrying capacity *k* using the (21), which estimates the total population of deer in Kansas (white tailed and mule deer combined) as 707,000. Thus, dividing this number by the number of cells yielded a value of *k* = 1, 095. Accounting for population fluctuation, we considered *k* values that differ from 1, 095 by 5%. Namely, we set *k* ∈ {1, 095, 1, 040, 1, 149}. We determined the possible transmission coefficients were drawn from published alternatives β ∈ {0.49, 0.8} reflecting different transmission assumptions in Potapov et al. (22). We estimated mortality rates μ from various survival estimates for infected deer (6) and considered μ ∈ {0.73, 0.93, 1.19}. Since direct estimates of ϵ and τ are limited, we selected ϵ ∈ {0.002, 0.003} and τ ∈ {0.85, 1.4} to satisfy stability conditions based on Maji et al. (23).

In each trial, upon initialization with data from the suitable starting year *t*_0_, for each combination of the parameter values considered above, we integrated the model (5) forward in time using standard numerical integration methods such as the standard Runge-Kutta 4-step method (24) with an integration time step corresponding to 1 day of simulated time, to obtain simulated infection values for each year in the validation window. Throughout the numerical integration process, the migration matrix was assumed to be constant. This grid-search resulted in a total of 5 × 3 × 2 × 3 × 2 × 2 = 360 integrations for each of the 50 trials. For each trial, the optimal parameter configuration was deemed to be the tuple (r, k, β, μ, ϵ, τ) that maximized model performance metrics (described below) over the validation window. For a given starting year *t*_0_, the overall optimal parameter configuration for the model (5) was estimated by computing the mode of each of the optimal parameter values across the 50 trials.

#### 2.2.6 Model evaluation

For each year *t* in the validation window, observed infection numbers were binarized at the cell level to form $I_{obs}^{\#}\left(t\right)\in {\mathbb{R}}^{646}$ for each *t* in the validation window. For each combination of parameter values in our search grid, the model (2) was initialized and integrated forward to obtain the infection intensity, defined to be the predicted number of infected cervids, in each cell for each *t* in the validation window. The infection intensities at time *t* were then normalized to be in the interval [0, 1] to obtain the vector $I_{sim}^{n}\left(t\right)$, which were interpreted as confidence scores for infection presence. This allowed us to evaluate our model as a binary classifier.

In each trial, we assessed the performance of our predictions $I_{sim}^{n}\left(t\right)$ against $I_{obs}^{\#}\left(t\right)$ by computing the Area Under the Curve (AUC) of the Receiver-Operating Characteristic (ROC) curve for each simulation year in our validation window. The optimal parameter configuration was determined to be the configuration that maximized the overall AUC values across the validation window, and the corresponding model with the optimal parameters was chosen to be the champion model of the trial. By averaging the AUC values across all 50 trials for a given starting year, we assessed the average performance of our model and produced uncertainty envelopes for the predicted *I*(*t*) in the form of error bars around the model performance (AUC) values for each year in the simulation window.

#### 2.2.7 Direction and rate of spread

**Figure 2:**
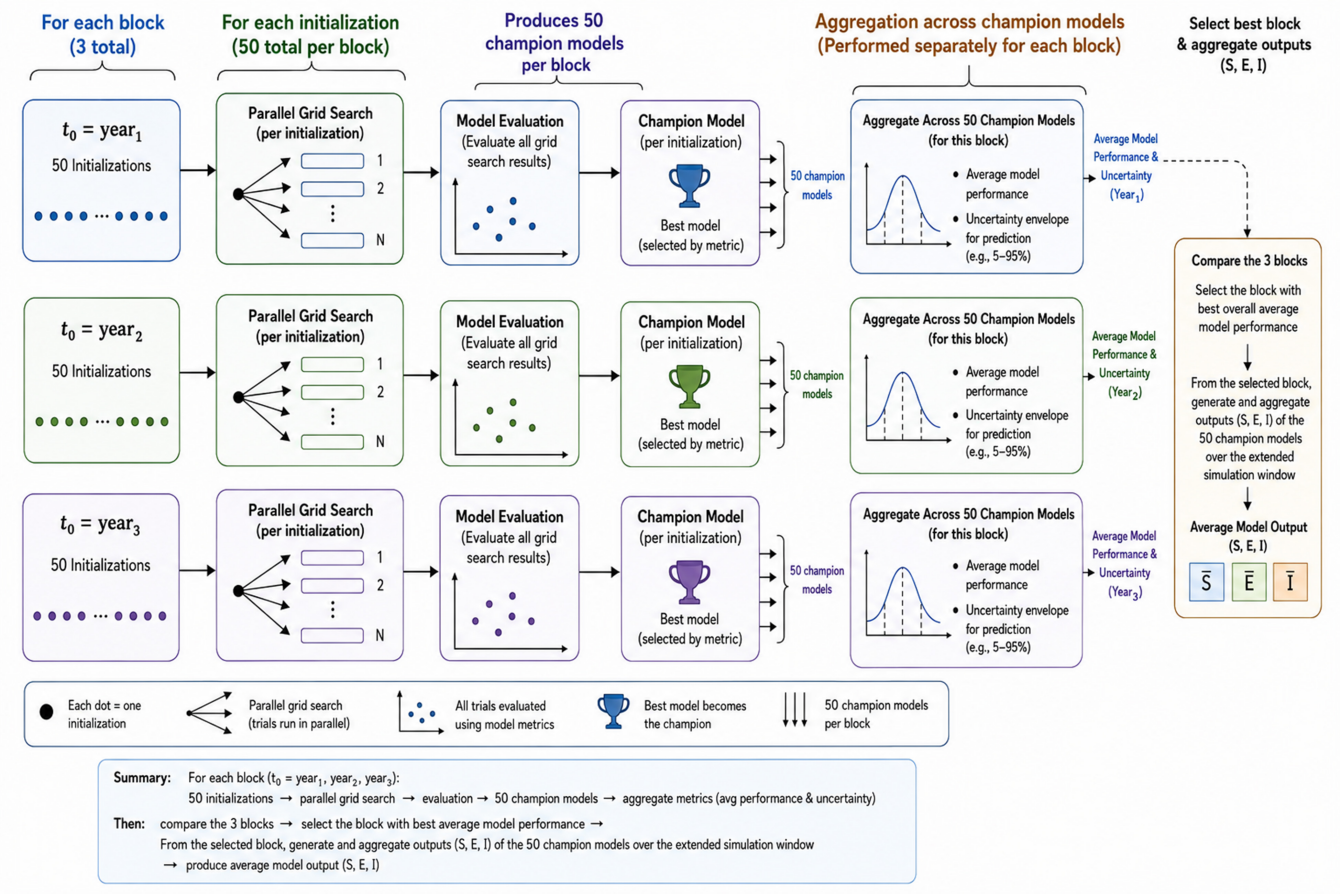
Model evaluation and aggregation pipeline.

To estimate the direction and rate of spread of disease, we extended the simulation window by setting *t*_1_ = 2, 025. We picked the starting year *t*_0_ (among the three choices) that resulted in the highest overall AUC across space and time. We initialized each of our 50 champion models with the data from this year and integrated them over the extended simulation window. We then averaged the resulting infection profiles to obtain an average estimate of the model's predictions. The model evaluation and aggregation pipeline is illustrated diagrammatically in Figure 2. Once the mean infection profiles were generated, we determined the rate and direction of disease spread at two scales. First, at a zonal scale, i.e., one of the five CWD surveillance zones established by KDWP in Kansas, and second, at the neighborhood level of cells, i.e., migration of infection from each cell to its neighbors. At the zonal level, infection dynamics were characterized by computing a weighted centroid value that proportionally reflected the level of infection present in a given zone and tracking its displacement over time. At the local level of cells, we utilized the migratory spatial component of our model to capture the instantaneous local direction and rate of infection spread from each cell to its neighbor.

Zonal analysis: given the simulated infection numbers $I_{sim}\left(Z,t\right)\in {\mathbb{R}}^{646}$ in year *t* over zone *Z*, we normalized the infection vector to obtain $I_{sim}^{n}\left(Z,t\right)\in {\mathbb{R}}^{646}$. We then placed a point-mass on each cell where $I_{sim}^{n}\left(Z,t\right)>0$ with the weight of the point-mass set equal to the value of this vector in that cell. This allowed us to calculate the weighted centroid in year *t* over zone Z as the center of mass of the resulting point configuration, given by:


$$\begin{array}{l} C\left(Z,t\right)=\frac{\sum_{i\in Z}I_{\text{sim}}^{n}\left(Z,t\right)\left[i\right]\cdot \left(i_{x},i_{y}\right)}{\sum_{i\in Z}I_{\text{sim}}^{n}\left(Z,t\right)\left[i\right]} \end{array}$$ (6)

where (*i*_*x*_, *i*_*y*_) denotes the center of cell *i*. We followed the displacement of this centroid, defined in Equation 6, across the simulation window to gain an intuitive picture of the dominant direction of infection spread within each zone. We also computed the velocity of these centroids to obtain an estimate of the average rate at which infection is progressing at the zonal level.

Neighborhood analysis: at the more granular level of cells, we used the migration matrix to estimate instantaneous flow of infection across cell boundaries. Since simulated infection numbers given by (5) is presented at a continuous scale, we determined annual classification thresholds κ_*t*_ that allowed us to only classify a cell as being infectious during year t if its normalized infection value falls above this threshold κ_*t*_. More precisely, we computed κ_*t*_ by solving the optimization problem in Equation 7 seeking to maximize the balanced accuracy of our model given by:


$$\begin{array}{l} \kappa_{t}:=\text{arg}{\text{max}}_{k\in \left[0,1\right]}\frac{r\left(I_{\text{sim}}^{n}\left(k,t\right)\right)+r\left(1-I_{\text{sim}}^{n}\left(k,t\right)\right)}{2} \end{array}$$ (7)

where *r*(−) is the recall of our model viewed as a binary classifier, and $I_{sim}^{n}\left(k,t\right)\in {\mathbb{R}}^{646}$ is obtained by thresholding the predicted infection values in year *t* at level *k*. To enable predictions at a granular time steps, we determined κ as the median of the annual thresholds κ_*t*_ across the validation window. Upon determining a suitable κ, we set $I_{sim}^{n}\left(\kappa ,t\right)$ to be the normalized infection numbers thresholded at level κ. Finally, we associated to each neighbor *j* of cell *i* a direction vector β_*ij*_ determined by the relative position of cell *j* to cell *i*. These unit vectors are drawn from the following set representing the eight possible migratory directions:


$$\begin{array}{l} B=\left\{\pm e_{1},\pm e_{2},\frac{1}{\sqrt{2}}\left(\pm e_{1}+\pm e_{2}\right)\right\} \end{array}$$

where $e_{1},e_{2}\in {\mathbb{R}}^{2}$ are canonical basis vectors. This enabled us to determine a local flow vector *v*_*i*_ for each cell *i* as a weighted sum of directional components:


$$\begin{array}{l} v_{i}=\underset{j\sim i}{\sum }m_{j\leftarrow i}I_{\text{sim}}^{n}\left(\kappa ,t\right)\left[i\right]\beta_{ij} \end{array}$$

where the quantity $m_{j\leftarrow i}I_{sim}^{n}\left(\kappa ,t\right)\left[i\right]$ is the number of infectious cervids that migrate from cell *i* to a neighboring cell *j* during the discrete time-step *t* → *t* + 1. The rate of infection spread from cell *i* can be inferred from the magnitude of *v*_*i*_, while the direction of spread at the cell-level is captured by its polar angle.

## 3 Results

### 3.1 Model performance

**Figure 3:**
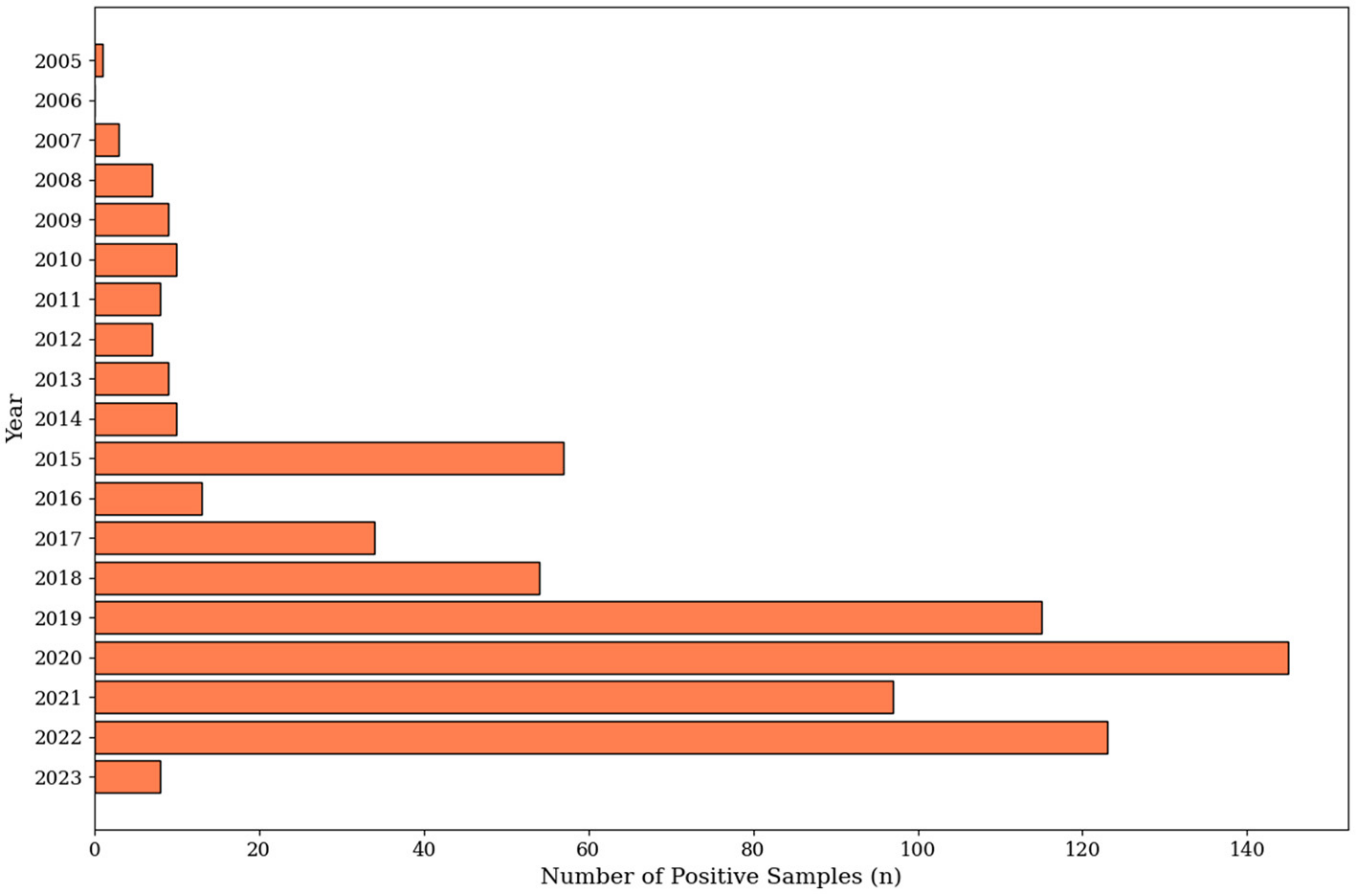
Annual number of 20 km^2^ grid cells in Kansas in which positive chronic wasting disease samples were detected between 2005–2023.

**Figure 4:**
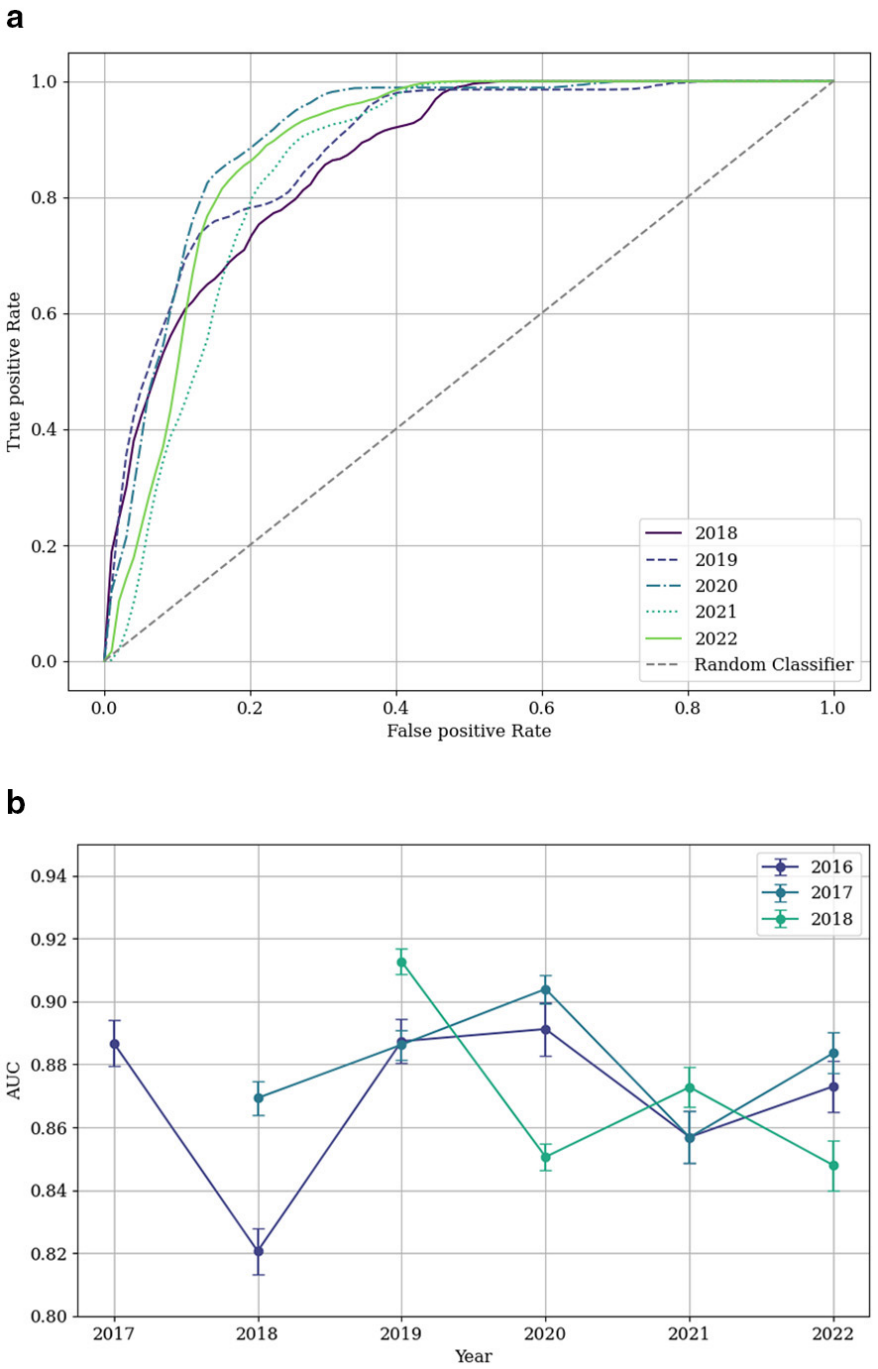
**(a)** Receiver-operating characteristic (ROC) curves for model-2, evaluated as a binary classifier for each simulation year from 2018–2022. **(b)** Mean area under the curve (AUC ± SE), averaged across 50 simulation trials for each year of the prediction window, under different model initialization starting year (*t*_0_ = 2016, 2017, and 2018).

Examination of the annual number of 20 km^2^ cells with positive CWD detection in Kansas revealed a pronounced expansion during 2018–2022 (Figure 3), yielding candidates *t*_0_= 2016, 2017 and 2018 for the starting year of our simulation, with 2022 serving as the terminal year of our validation window. Model discrimination, evaluated over the validation window also yielded strong performance across all three starting years, with the overall AUC (averaged across the 50 trials) maximized for the starting year *t*_0_ = 2017, exhibiting values ranging from 0.85 to 0.90 and a mean of 0.88 (Table 2); indicating that the optimized model conforms satisfactorily with the spatiotemporal trends in the underlying data without overfitting. Moreover, the confidence envelopes along with the average AUC values (Figure 4b, Table 2) attained by the model across all initializations indicate stable performance, with the largest standard deviation from the mean by only 0.8%. The optimal parameter configuration for the model (5) was then estimated by computing the mode of each parameter across all 50 models, resulting in (r, k, β, μ, ϵ, τ) = (2.5, 1,040, 0.49, 1.19, 0.002, 0.85). The ROC curves, averaged across the 50 champion models (Figure 4a), indicated robust performance across all years included in the simulation window (2018–2022), notably achieving greater than 80% specificity and 80% sensitivity at low classification thresholds (Table 2, Figure 4a).

**Table 2:** AUC values and their standard deviation of model (2) for each year in the validation window for each choice of starting year *t*_0_.

*t*_0_	2017	2018	2019	2020	2021	2022	Overall
2016	0.8867 ±0.007	0.8205 ± 0.007	0.8873 ± 0.007	0.8912 ± 0.008	0.8569 ± 0.008	0.8730 ± 0.008	0.8692 ± 0.004
2017	—	0.8692 ± 0.005	0.8862 ± 0.005	0.9039 ± 0.005	0.8567 ± 0.008	0.8835 ± 0.006	0.8799 ± 0.002
2018	—	—	0.9126 ± 0.004	0.8505 ± 0.004	0.8728 ± 0.006	0.8479 ± 0.008	0.8709 ± 0.003

**Figure 5:**
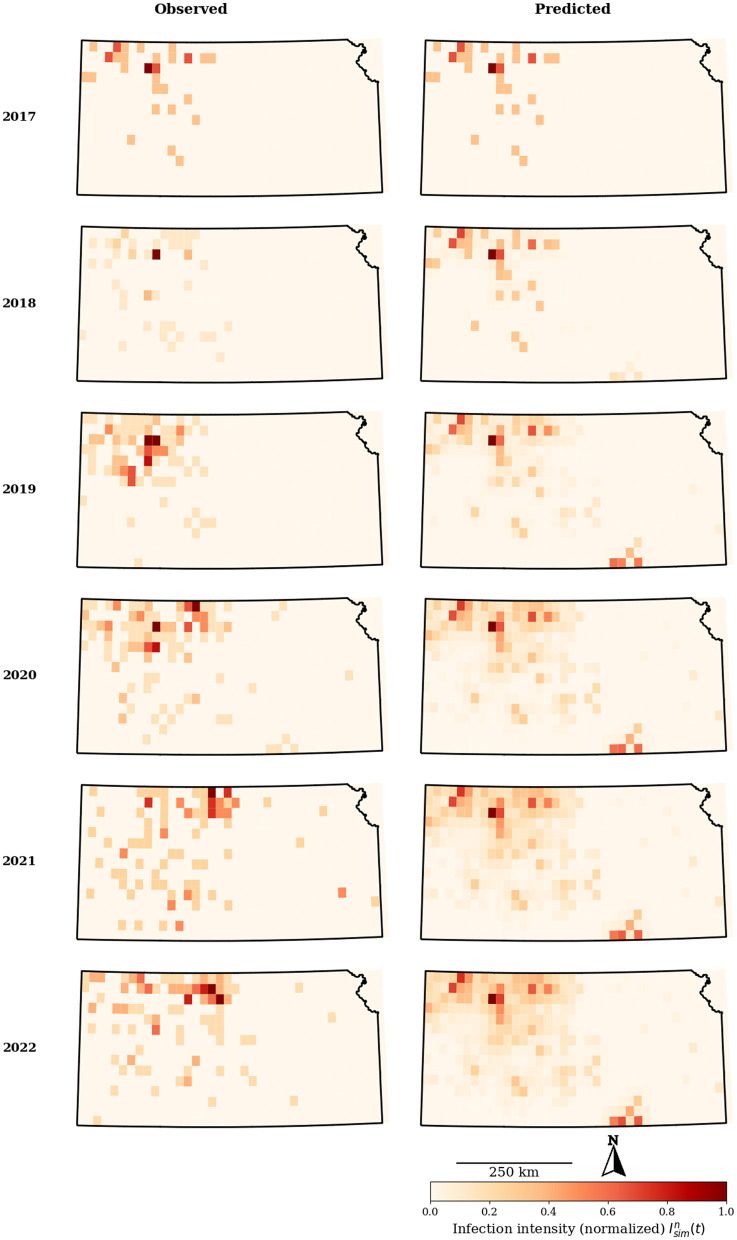
Observed vs. predicted chronic wasting disease infection presence within 20 km^2^ grid cells across Kansas over the years 2018 – 2022 post model normalization.

**Figure 6:**
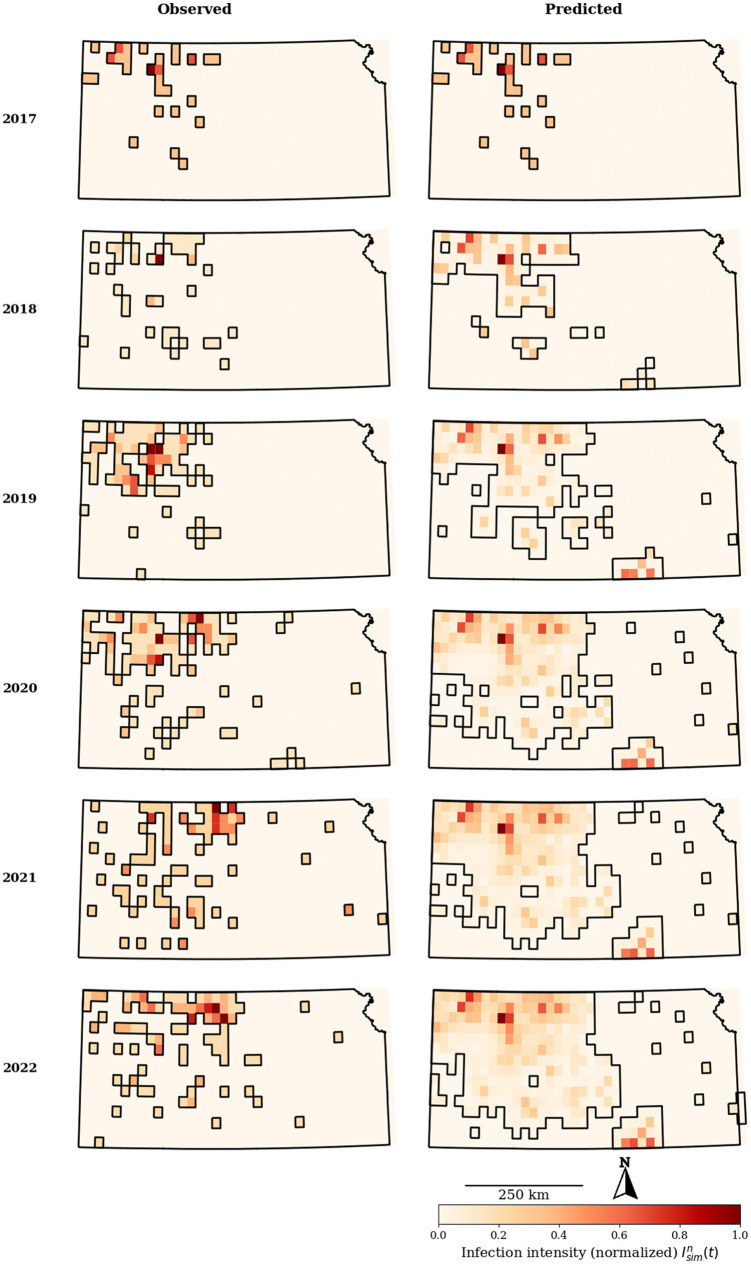
Spatial boundaries of observed and predicted chronic wasting disease infection presence within 20 km^2^ grid cells across Kansas from 2018–2022 following model normalization.

Importantly, in each trial, the model was initialized using prevalence data from a single year and was not fit to subsequent years, yet it closely reproduced the observed spatial patterns (Figures 5, 6). This demonstrated that the deterministic temporal structure, coupled with empirically informed constant-rate migration, is sufficient to recover the dominant spatiotemporal signal in the surveillance data.

### 3.2 Spatiotemporal reconstruction of infection

The estimated observed and predicted spread of infection (averaged across all 50 champion models with *t*_0_ = 2017) across Kansas over the simulated years is presented in Figure 5, which visually confirms satisfactory performance of the optimized model. The model is successful in capturing the broad migratory trends in the observed surveillance data, while also correctly forecasting infectious sites that only materialize in the observed data years later. The northwestern region remained a stable area of high-infection across all years, while the north-central and south-central regions exhibited progressive range expansion. In contrast, the eastern zone showed comparatively sparse detections and lower simulated prevalence of CWD, consistent with limited sampling density and later arrival of the disease front in this region.

Additionally, Figure 6 identifies the boundaries of grid cells with CWD infection across Kansas and reveals potential trends in the presence of infection in grid cells that have not been previously identified. Notably, two distinct and persistent clusters appear in the observed as well as predicted data through the simulation window.

### 3.3 Zonal estimates of rate and direction of spread

**Figure 7:**
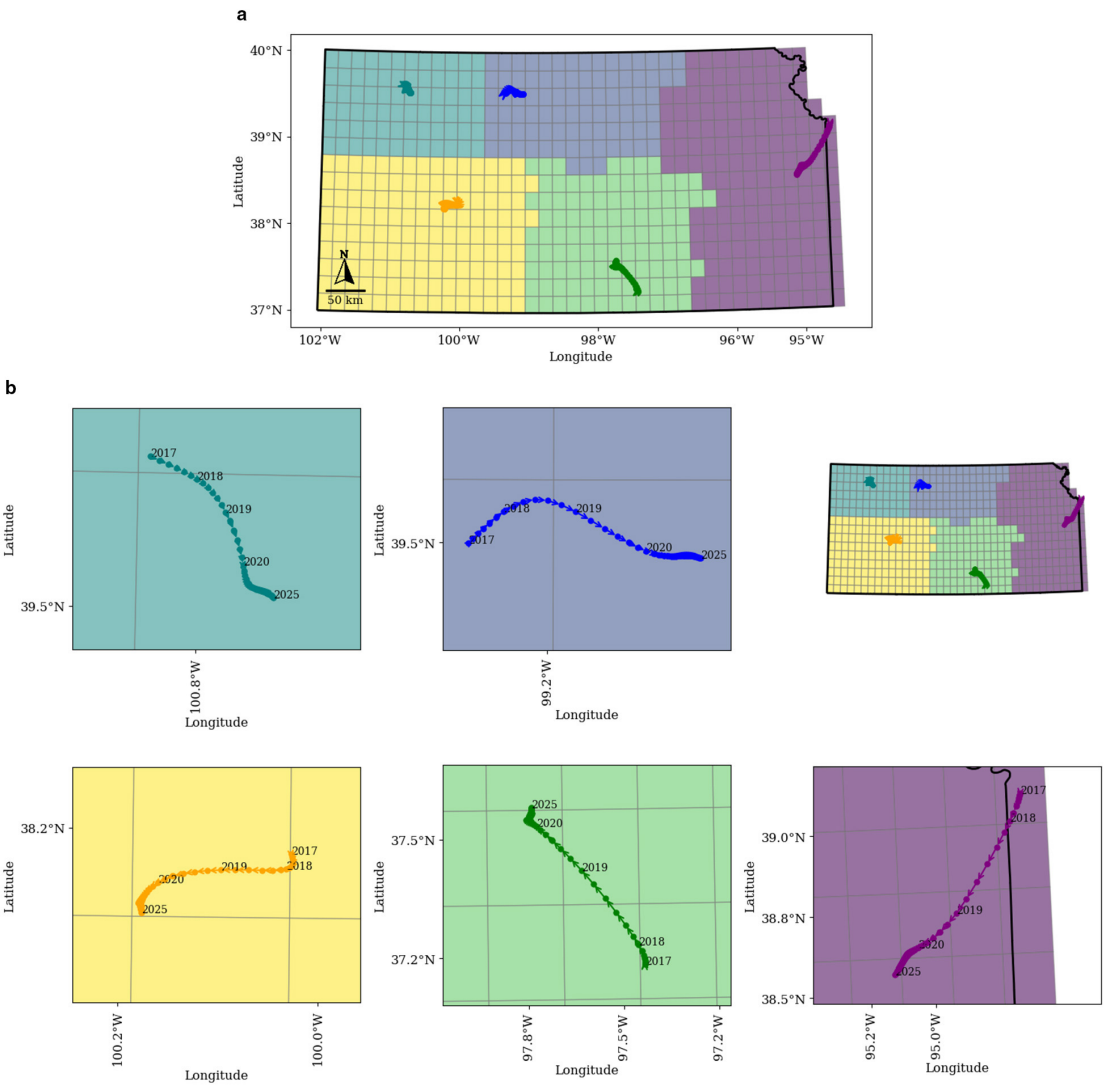
**(a)** The overall progression of chronic wasting disease infection status over the years 2018–2025 within each of the five zones with infectious-centroids, calculated over 2-month time steps. **(b)** A closer view of the overall geographic progression of chronic wasting disease infection status over the years 2018–2025 for each of the five management zones, with weighted centroids calculated over 2-month time steps, and years indicated in the plot for the period 2018–2022 along with the terminal year 2025.

Weighted centroids of normalized simulated prevalence were computed for each zone at 2-month intervals from 2018 to 2025 (Figures 7a, b). The resulting centroid trajectories revealed heterogeneous movement of infection status across regions, with a dominant northwest-to-southeast progression at the statewide level. Average zonal velocities (Table 3) quantify this heterogeneity: besides the eastern zone, the south-central zone exhibited the greatest overall rate of spread, followed by the eastern and north-central zones, whereas the northwestern zone showed minimal centroid displacement. The nuances pertaining to the large displacement profiles in the eastern zone are discussed in length in Section 4.

**Table 3:** Average rate of spread of infection (averaged movement of weighted centroids) within different chronic wasting disease management zones in Kansas.

Zone	Horizontal rate (km/year)	Vertical rate (km/year)	Overall rate
Northwest	1.08	−1.18	1.60
Southwest	−2.40	−0.96	2.59
North central	3.20	−0.13	3.21
South central	−3.79	5.15	6.40
East	−6.04	−8.80	10.68

Temporal changes in centroid direction further revealed complex dynamics (Figures 7a, b). In the south-central zone, centroids initially shifted southeastward (2018–2019), reflecting the development of a southern hotspot, before reversing toward the northwest as infection diffused eastward and northern sources continued to exert influence. In the eastern zone, centroid movement was predominantly southwestward, capturing spillovers from the south-central region. These patterns indicate that regional spread of CWD is not uniform but is shaped by localized sources, sinks, and potentially inter-zonal connectivity through movement corridors such as rivers.

### 3.4 Local estimates of direction and rate of spread

**Figure 8:**
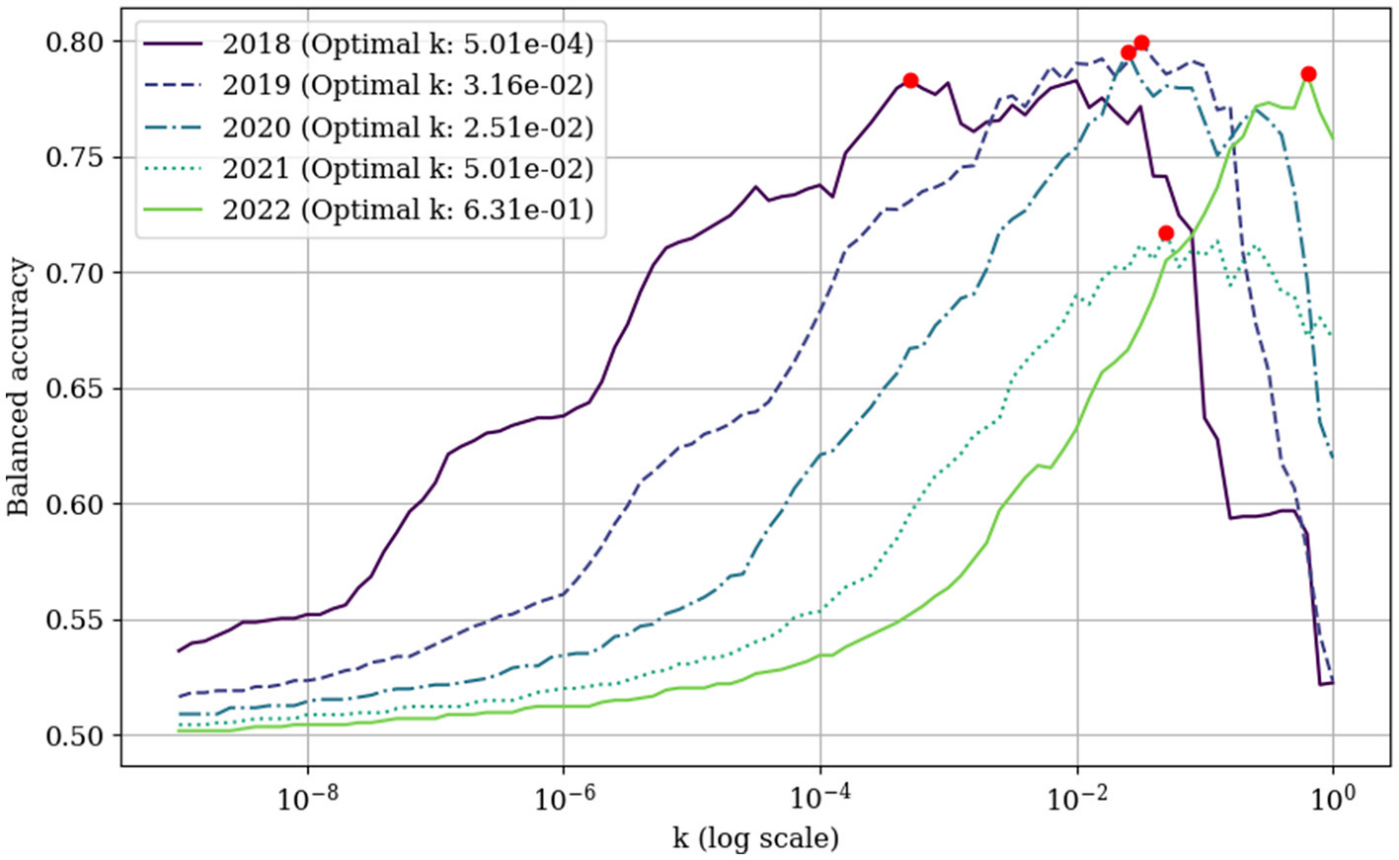
Balanced accuracy plots to determine the classification threshold κ for local migratory analysis.

**Figure 9:**
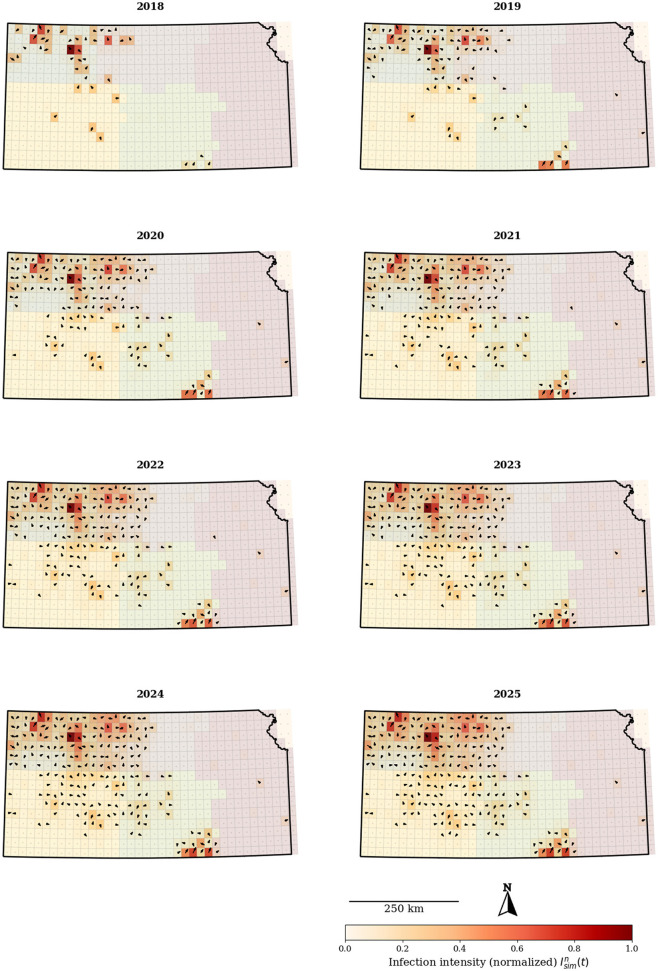
Local infection spread for years 2018–2025 across all zones, with colors indicating the normalized infection intensity, and the arrow indicating the overall local direction of flow of infection from one cell to its neighbor.

The optimal classification thresholds κ_*t*_ (Table 4) obtained through a line search (Figure 8), exhibit satisfactory performance. Taking the median of all the κ_*t*_ values over the validation window yielded a uniform classification threshold of κ = 0.0316. Local flow vectors computed from the migration matrix and directional basis produced annual flow fields for 2018–2025 (Figure 9). The vector magnitude ||*v*_*i*_|| represents the rate of spread of infection from a given cell to its neighbors (Tables 5, 6, Supplementary File 1), while the arrow's orientation centered at each cell indicates the direction in which infection spreads. The length of the arrow is proportional to the rate at which infection from a given cell is migrating.

**Table 4:** Estimated classification threshold κ_*t*_ for each year *t* = 2018–2022, and the corresponding balanced accuracy metric.

Year	2018	2019	2020	2021	2022
Threshold *κ_*t*_*	5.01 × 10^−4^	3.16 × 10^−2^	2.51 × 10^−2^	1.21 × 10^−1^	6.31 × 10^−1^
Balanced accuracy	0.78	0.80	0.79	0.71	0.78

**Table 5:** Estimated local infection spread rates (km per 60-day interval) at the beginning of each year from 2018–2025 for the 10 counties with the highest predicted rates of spread.

Country	Zone	*2018*	*2019*	*2020*	*2021*	*2022*	2023	2024	2025
Sumner	SC	0.0304	0.1590	0.3591	0.7136	1.3948	2.8606	5.4964	10.3834
Cowley	SC	0.0275	0.1298	0.284	0.5596	1.095	2.2645	4.4004	8.4081
Rawlins	NW	0.121	0.1405	0.3048	0.6025	1.1529	2.3166	4.3873	8.2039
Sherman	NW	0.1006	0.1091	0.2563	0.5338	1.0454	2.1513	4.1796	8.0669
Sheridan	NW	0.0611	0.0737	0.173	0.3602	0.7138	1.4846	2.9013	5.6037
Decatur	NW	0.0772	0.0774	0.1752	0.3569	0.6842	1.3846	2.6676	5.1409
Cheyenne	NW	0.0081	0.0327	0.1183	0.265	0.5418	1.163	2.3472	4.7126
Graham	NC	0.0089	0.0351	0.1171	0.259	0.5335	1.1502	2.3177	4.6196
Phillips	NC	0.0091	0.0445	0.1227	0.2582	0.5226	1.112	2.2156	4.3614
Norton	NW	0.0399	0.0549	0.1285	0.2601	0.5067	1.0379	2.003	3.8248

Years shown in italic denote validation years for model 2.

**Table 6:** Estimated local infection spread rates (km per 60-day interval) at the beginning of each year from 2018–2025 for the 10 grid cells with the highest predicted spread rates.

Country	Zone	Cell id	*2018*	*2019*	*2020*	*2021*	*2022*	2023	2024	2025
Sumner	SC	25	0.0304	0.159	0.3591	0.7136	1.3948	2.8606	5.4964	10.3834
Cowley	SC	27	0.0275	0.1298	0.284	0.5596	1.095	2.2645	4.4004	8.4081
Rawlins	NW	498	0.121	0.1405	0.3048	0.6025	1.1529	2.3166	4.3873	8.2039
Sherman	NW	419	0.1006	0.1091	0.2563	0.5338	1.0454	2.1513	4.1796	8.0669
Rawlins	NW	537	0.0968	0.1169	0.2438	0.4767	0.9103	1.8266	3.4542	6.4461
Sumner	SC	24	0.0325	0.1375	0.2779	0.507	0.9209	1.7649	3.2187	5.834
Sheridan	NW	428	0.0611	0.0737	0.173	0.3602	0.7138	1.4846	2.9013	5.6037
Rawlins	NW	499	0.0736	0.0918	0.206	0.4076	0.7754	1.5514	2.9378	5.5269
Sheridan	NW	427	0.0167	0.033	0.1007	0.248	0.5479	1.2482	2.6137	5.3632
Sherman	NW	457	0.0127	0.0264	0.096	0.2448	0.5397	1.2244	2.5603	5.2668

Years shown in italic denote validation years for model 2.

Beyond the local dynamics, Figure 9 further reveals how neighborhood-level movements aggregate to generate the broader statewide trends. For instance, in Figure 9, the overall spread of the infection can be observed to be from the northwestern zone to the north and southcentral zones, and this spread is distinct from the local dynamics seen in the southern part of the state.

The neighborhood-level plots allow us to identify regions of higher rates of spread indicated by long migration vectors. Several cells in the southcentral zone along the southern border, particularly in Sumner and Cowley counties, exhibit high rates of northeastward spread into the eastern zone of Kansas. Additional regions with high rates of infection spread are found across the northwestern region, particularly near the northern border, along Decatur, Norton and Rawlin counties where the predominance of southward-oriented arrows suggests possible infection incursions into these counties from southern Nebraska. Along the western border, the vectors are aligned facing east, indicating a second potential input of infection from Northern Colorado. In the northcentral zone, areas of intensified migration with high rates of spread are concentrated once again along the northern border; however, the orientation of the vectors indicate movement northward into Nebraska.

## 4 Discussion

The spatiotemporal model developed in this study performed well and enabled quantification of both the direction and rate of CWD spread across Kansas. With a mean AUC of 0.880 across study years, equivalent to a predictive accuracy of 88%, the model demonstrates strong potential as both a retrospective and predictive tool for CWD management in Kansas and potentially elsewhere if appropriately applied. Empirical analyses of observed CWD spatiotemporal patterns in Kansas are consistent with the broader spatial dynamics captured by the model and our previous evaluations of the data (3, 4). Surveillance data indicate steady expansion of disease from the northwestern region toward the southeast over time; the model reproduces this trend with high fidelity (Figure 5).

In addition to accurately reproducing observed patterns, the model demonstrates clear predictive capacity. In the south-central zone (Figure 5), it forecasted an infection hotspot along Kansas's southern border 2 years prior to official detection in 2020. Similarly, mild infection activity predicted in the eastern corridor in 2019 was later confirmed through field observations in 2021. These forward-looking examples underscore the model's predictive strength.

The boundaries of infectious mass for observed and predicted infection intensities (number of infected cervids in a given cell) across Kansas are illustrated in Figure 6. Examination of their evolution reveals two persistent clusters: one originating in the northwest and another along the southern border. These clusters remain separated by a diagonal, infection-free corridor evident in both simulated and observed data. This configuration suggests that CWD spread in Kansas is more complex than a single northwest-to-southeast migratory front. Instead, the evidence supports bifurcated spread along two distinct pathways: one extending southward and southeastward from the northwestern and north-central regions, and another emerging independently along the southern border. Although the northwest-to-southcentral migration is substantial, zonal movement estimates indicate that the northwestern zone itself exhibits limited net displacement (1.60 km/year overall), reinforcing its role as a persistent source region rather than a rapidly advancing front. In contrast, the south-central zone demonstrates substantially greater centroid movement (6.40 km/year overall), consistent with interaction between the northwestern and southern-border transmission strands. This southern-border strand was not identified in prior occupational or spatiotemporal modeling studies using the same dataset (3, 4).

Additional support for this dual-front pattern comes from zonal weighted centroid shifts (Figures 7a, b) and cell-level analyses (Figure 9). In the south-central zone, centroids initially shifted southeast (2018–2019), likely responding to new detections at the southern border. As this new southern strand migrated eastward, centroid movement reoriented toward the central regions of the zone reflecting the southward expansion of the northwestern front. The magnitude of this movement is reflected in the strong horizontal (−3.79 km/year) and vertical (+5.15 km/year) displacement components observed in this zone. In the eastern zone, centroids shifted sharply southwestward, with large horizontal (−6.04 km/year) and vertical (−8.80 km/year) components yielding the highest overall displacement estimate (10.68 km/year). However, as discussed below, these high rates likely reflect limited sampling density rather than sustained directional expansion. Cell-level plots (Figure 9) further confirm the elevated eastward spread along the southern border alongside sustained expansion of the northwestern cluster. Together, these findings indicate that CWD spread in the south-central and eastern regions is driven by two transmission pathways.

The southern incursion may have resulted from movement of migrating cervids in northern Oklahoma, possibly via the Osage National Reserve, or from improper disposal of infected carcasses transported from northwestern Kansas. Both possibilities warrant further investigation, as clarifying localized movement patterns could inform targeted prevention and control strategies.

Beyond the south-central dynamics, clear patterns also emerge in the northwestern and north-central regions. Infection centroids in the northwestern zone remain concentrated near Decatur and Sheridan Counties (Table 3; Figure 7b), aligning with the highest observed and simulated infection intensities statewide (Figure 5). Zonal spread rates indicate minimal internal movement in this region (horizontal 1.07 km/year; vertical −1.17 km/year), consistent with persistent hotspots from 2017–2022. In contrast, the north-central zone shows moderate net displacement (3.19 km/year overall), primarily driven by eastward movement (3.19 km/year). Internal KDWP and USDA reports suggest CWD in Decatur County may have originated from an infected elk herd later unaccounted for, potentially accelerating long-term establishment given the high transmissibility of elk prions to white-tailed deer. Cell-level analyses (Figure 9) indicate these hotspots likely function as sources, seeding adjacent areas and contributing to southeastward spread. Notably, six of the seven cells with the highest infection rates (Table 6) in the northwestern zone correspond to Sheridan and Rawlins counties, bordering Decatur County and southern Nebraska.

Local analyses (Figure 9, Supplemental File 1) reveal consistent southward movement along the northern border and eastward movement along the western border, suggesting possible dual incursion into Kansas from southern Nebraska and eastern Colorado. Supporting this, CWD has been documented in unit 13 of southern Nebraska bordering northwestern Kansas, and in northeastern Colorado as of 2022, also bordering the northwestern zone (25, 26). Concurrently, migration rates in the north-central zone show northward flow into Nebraska from Smith and Phillips counties at approximately 6 km every 2 months, suggesting bidirectional transmission across state lines. Monitoring this corridor could strengthen management efforts.

In the southwestern zone, zonal analyses indicate gradual westward shift (horizontal −2.36 km/year) with relatively limited vertical displacement, yielding a moderate overall rate (2.55 km/year). Despite this, infection has not yet reached the far southwestern boundary. Spread rates here remain among the lowest of the affected zones. Targeted check stations along the slow-moving infection boundary (Figure 6) could facilitate early detection and mitigation. In contrast, although the eastern zone exhibits the highest centroid displacement estimate, cell-level rates suggest local migration remains slow, supporting the interpretation that zonal velocity estimates may be inflated by sparse sampling. Expanded surveillance would likely improve movement estimates in this region.

Despite the strengths of this framework, limitations remain. Chief among them is the absence of environmental and explicit host-factor (age, sex) inputs, which are known to influence deer movement and disease dynamics. Incorporating habitat-level land cover, land use, soil, and elevation data would likely improve the representation of the rate and direction of CWD spread. Our prior work has identified the influence of land cover, soil, and elevation on CWD in the state. Future extensions should dynamically integrate these environmental and host-factor variables at appropriate spatial scales to enable more comprehensive modeling of disease progression.

## 5 Conclusion

This study delivers a quantitative, multi-scale characterization of the rate and direction of CWD spread across Kansas. By uniting mechanistic disease dynamics with empirically informed migration, the framework reveals dual-front propagation, identifies persistent sources and high-flux corridors, and provides potential early-warning signals of emerging hotspots. In systems where conventional interventions are limited, such spatially explicit, predictive tools are critical for designing targeted surveillance, prioritizing management actions, and mitigating the continued expansion of chronic wasting disease.

## Data Availability

The data used in this publication can be made available for reasonable use in research by the author at the discretion of University of Missouri and Kansas Department of Wildlife and Parks.

## References

[1] Williams ES. Chronic wasting disease. Vet Pathol. (2005) 42:530–49. doi: 10.1354/vp.42-5-530 16145200

[2] CDC. About Chronic wasting disease. Atlanta, GA: CDC (2026). Available online at: https://www.cdc.gov/chronic-wasting/about/?CDC_AAref_Val=https://www.cdc.gov/prions/cwd/occurrence.html (Accessed May 8, 2026)

[3] Davis AJ Hesting S Jaster L Mosley JE Raghavan A Raghavan RK. Spatiotemporal occupancy patterns of chronic wasting disease. Front Vet Sci. (2024) 11:1492743. doi: 10.3389/fvets.2024.1492743 39634764 PMC11615082

[4] Bishop RC. The economic impacts of chronic wasting disease (CWD) in Wisconsin. Human Dimensions of Wildlife. (2004) 9:181–92. doi: 10.1080/10871200490479963

[5] Chiavacci SJ. The economic costs of chronic wasting disease in the United States. PLoS ONE. (2022) 17:e0278366. doi: 10.1371/journal.pone.0278366 36480524 PMC9731425

[6] Edmunds DR Kauffman MJ Schumaker BA Lindzey FG Cook WE Kreeger TJ . Chronic wasting disease drives population decline of white-tailed deer. PLoS ONE. (2016) 11:e0161127. doi: 10.1371/journal.pone.0161127 27575545 PMC5004924

[7] DeVivo MT Edmunds DR Kauffman MJ Schumaker BA Binfet J Kreeger TJ . Endemic chronic wasting disease causes mule deer population decline in Wyoming. PLoS ONE. (2017) 12:e0186512. doi: 10.1371/journal.pone.0186512 29049389 PMC5648191

[8] Jonathan DC David MW William FP Sonja AC. Improved predictions and forecasts of chronic wasting disease occurrence using multiple mechanism dynamic occupancy modeling. J Wildlife Manag. (2022) 86:e22296. doi: 10.1002/jwmg.22296

[9] Davis AJ Kirby JD Chipman RB Nelson KM Gilbert AT. Data-driven management—A dynamic occupancy approach to enhanced rabies surveillance prioritization. Viruses. (2021) 13:1795. doi: 10.3390/v13091795 34578376 PMC8472164

[10] Wang X Wang H Li MY. Modeling rabies transmission in spatially heterogeneous environments via θ-diffusion. Bull Math Biol. (2021) 83:16. doi: 10.1007/s11538-020-00857-1 33433727

[11] Kansas Department of Wildlife and Parks. Surveillance Zones Map. Available online at: https://www.ksoutdoors.gov/programs-services/controlling-wildlife-diseases/deer-chronic-wasting-disease#maps (Accessed June 13, 2026).

[12] Sharp A Pastor J. Stable limit cycles and the paradox of enrichment in a model of chronic wasting disease. Ecol Appl. (2011) 21:1024–30. doi: 10.1890/10-1449.1 21774409

[13] Denkers ND McNulty EE Kraft CN Nalls AV Westrich JA Hoover EA . Temporal characterization of prion shedding in secreta of white-tailed deer in longitudinal study of chronic wasting disease, United States. Emerg Infect Dis. (2024) 30:2118. doi: 10.3201/eid3010.240159 39320164 PMC11431932

[14] Mathiason CK Hays SA Powers J Hayes-Klug J Langenberg J Dahmes SJ . Infectious prions in pre-clinical deer and transmission of chronic wasting disease solely by environmental exposure. PLoS ONE. (2009) 4:e5916. doi: 10.1371/journal.pone.0005916 19529769 PMC2691594

[15] Pritzkow S. Transmission, strain diversity, and zoonotic potential of chronic wasting disease. Viruses. (2022) 14:1390. doi: 10.3390/v14071390 35891371 PMC9316268

[16] Johns CJ Mehl CH. A dynamic spatial model for chronic wasting disease in Colorado. J Data Sci. (2006) 4:21–37. doi: 10.6339/JDS.2006.04(1).221

[17] Engineering National Academies of Sciences and Medicine. State of Knowledge Regarding Transmission, Spread, and Management of Chronic Wasting Disease in U.S. Captive and Free-Ranging Cervid Populations. Washington, DC: The National Academies Press (2025). p. 62–80.

[18] White SH Del Rey AM Sánchez GR. Modeling epidemics using cellular automata. Appl Math Comput. (2007) 186:193–202. doi: 10.1016/j.amc.2006.06.126 32287494 PMC7127728

[19] Joly DO Samuel MD Langenberg JA Blanchong JA Batha CA Rolley RE . Spatial epidemiology of chronic wasting disease in Wisconsin white-tailed deer. J Wildlife Dis. (2006) 42:578–88. doi: 10.7589/0090-3558-42.3.578 17092889

[20] Green ML Kelly AC Satterthwaite-Phillips D Manjerovic MB Shelton P Novakofski J . Reproductive characteristics of female white-tailed deer (*Odocoileus virginianus*) in the Midwestern USA. Theriogenology. (2017) 94:71–8. doi: 10.1016/j.theriogenology.2017.02.010 28407863

[21] World Population Review. Deer population by state 2025. (2025).

[22] Potapov A Merrill E Pybus M Lewis MA. Chronic wasting disease: Transmission mechanisms and the possibility of harvest management. PLoS ONE. (2016) 11:e0151039. doi: 10.1371/journal.pone.0151039 26963921 PMC4786122

[23] Maji C Mukherjee D Kesh D. Deterministic and stochastic analysis of an eco-epidemiological model. J Biol Phys. (2018) 44:17–36. doi: 10.1007/s10867-017-9472-5 28988403 PMC5834997

[24] Burden RL Faires JD. Numerical analysis. 9th Edition. Boston: Brookscole. (2011) 259–3.

[25] Colorado Parks and Wildlife. Chronic Wasting Disease (2026). Available online at: https://cpw.state.co.us/activities/hunting/big-game/chronic-wasting-disease (Accessed May 8, 2026).

[26] Nebraska Game Parks. Chronic Wasting Disease (2026). Available online at: https://outdoornebraska.gov/conservation/conservation-challenges/wildlife-diseases/chronic-wasting-disease/ (Accessed May 8, 2026).

